# Multiple pancreaticobiliary fistulas combined with acute necrotizing pancreatitis: a rare complication of pancreatic extracorporeal shock wave lithotripsy

**DOI:** 10.1055/a-2361-1299

**Published:** 2024-08-08

**Authors:** Guangchao Li, Peng Wang, Limei Wang, Zhen Li, Rui Ji, Hongbo Ren, Ning Zhong

**Affiliations:** 1 91623 Department of Gastroenterology, Qilu Hospital of Shandong University, Jinan, China

A 54-year-old woman with a history of chronic pancreatitis and cholecystectomy 4 years prior presented with intermittent abdominal pain. Computed tomography (CT) revealed large pancreatic calcifications, main pancreatic duct (MPD) dilation, and pneumatosis in the bile and pancreatic ducts ( Fig. 1 ). She underwent pancreatic extracorporeal shock wave lithotripsy (ESWL) first, during which small stone fragments were expelled ( Fig. 2 ). However, her abdominal pain worsened, with low grade fever post-procedure. Laboratory tests and CT revealed acute necrotizing pancreatitis, extensive exudation and peripancreatic fluid collection, and impacted stones in the pancreatic head ( Fig. 3 ). Abdominal pain control proved difficult without analgesics. Enhanced CT suspected a connection between the MPD and the common bile duct (CBD) ( Fig. 3 , arrow). Further endoscopic retrograde cholangiopancreatography found two fistula openings near the major papilla, which proved to be bile and pancreatic duodenal fistulas. Pancreatography confirmed the presence of a pancreaticobiliary fistula, linking the distal CBD to the MPD ( Fig. 4 , Video 1 ). After clearing fragments, a 7-Fr × 9-cm single-pigtail plastic stent was placed and significantly improved her symptoms.

**Fig. 1:**
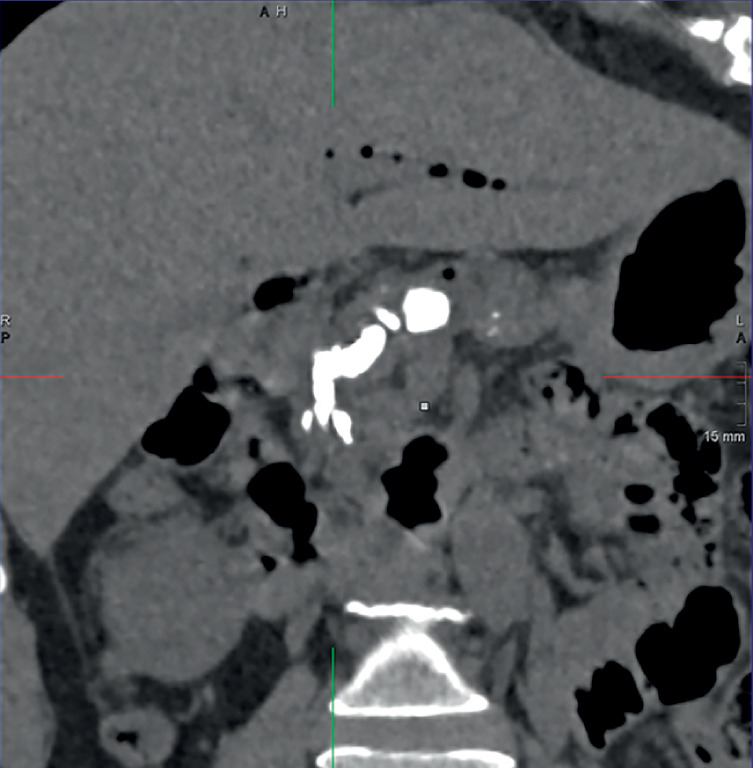
Computed tomography showed chronic pancreatitis with large calcifications, upstream main pancreatic duct dilation, and pneumatosis in the biliary and pancreatic ducts.

**Fig. 2:**
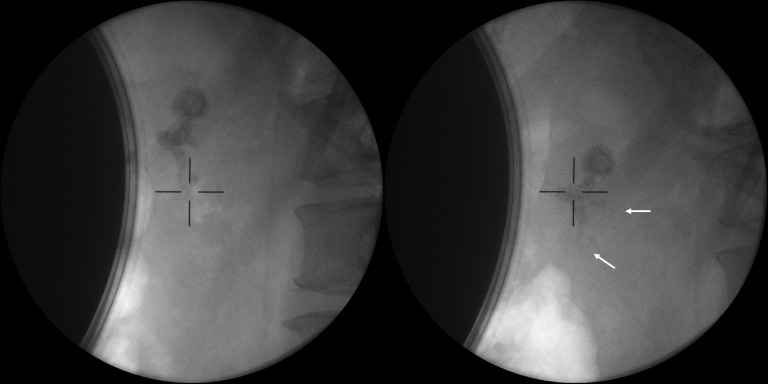
X-ray showed that stone fragments (arrows) were expelled after extracorporeal shock wave lithotripsy.

**Fig. 3:**
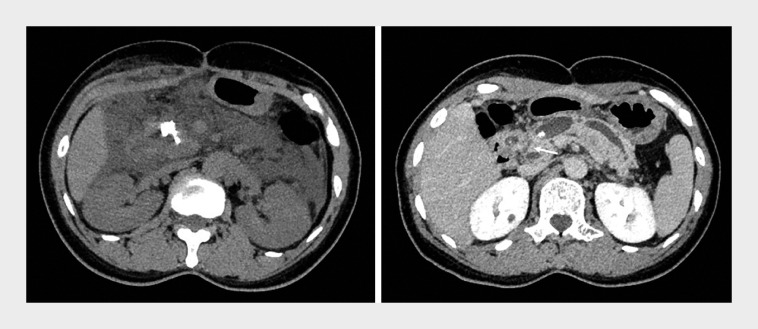
Computed tomography (CT) showed extensive exudation and peripancreatic fluid collection, and impacted stones in the pancreatic head (left). Enhanced CT revealed a suspected connection (arrow) between the common bile duct and the main pancreatic duct (right).

**Fig. 4:**
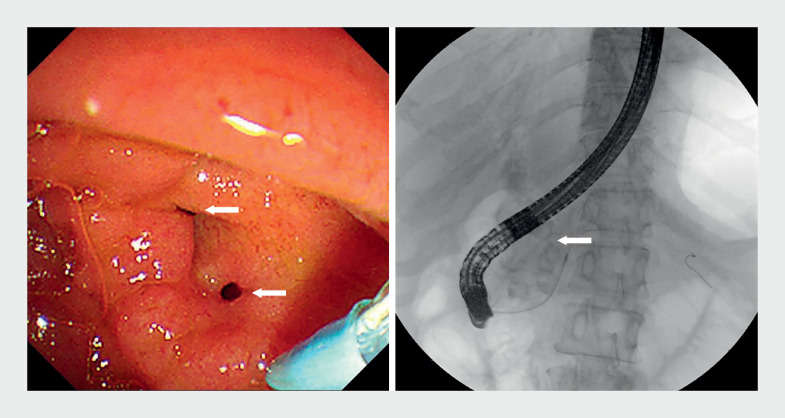
Endoscopic retrograde cholangiopancreatography showed two bile and pancreatic duodenal fistula openings (arrows) near the major papilla, and confirmed the presence of pancreaticobiliary fistulas.

Multiple pancreaticobiliary fistulas leading to pancreatitis after extracorporeal shock wave lithotripsy, and healing with endoscopic pancreatic stent drainage.Video 1

Remarkably, the pancreaticobiliary fistula had healed 2 months later. Pancreatoscopy revealed stenosis with no stones remaining in the MPD ( Fig. 5 , Video 1 ). However, as CT showed one stone remaining in the pancreatic parenchyma or branch duct, we placed two single-pigtail stents (7-Fr × 9-cm and 7-Fr × 8-cm) for better drainage.

**Fig. 5:**
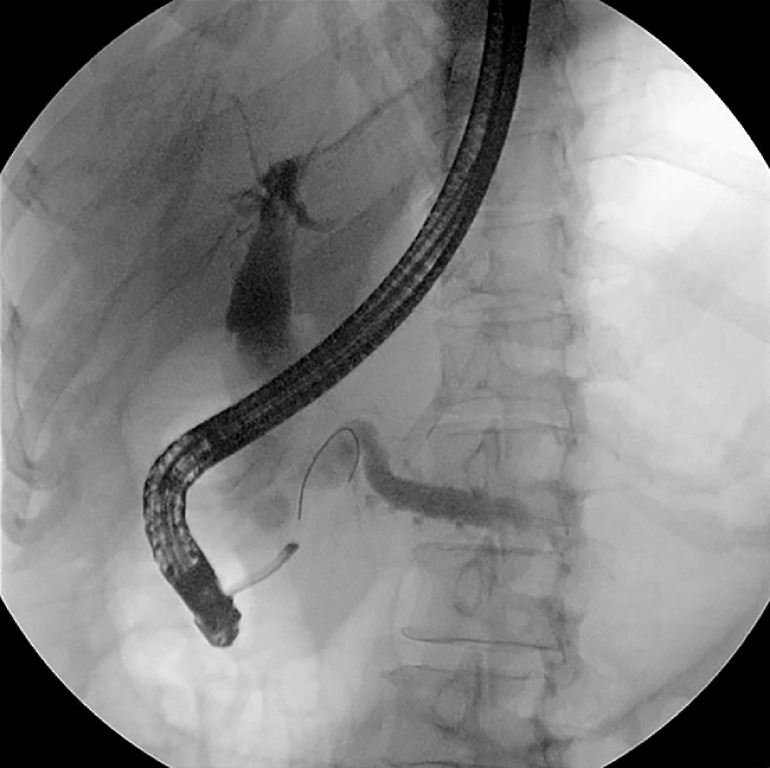
Endoscopic retrograde cholangiopancreatography showed that the pancreaticobiliary fistula had healed, with no stones remaining in the main pancreatic duct, but with stenosis and common bile duct dilation.

In the context of pancreatic ESWL, a minority of patients may experience acute pancreatitis with unknown etiology 1 . Multiple pancreaticobiliary fistulas as well as poor drainage is a rare etiology leading to post-ESWL pancreatitis, analogous to pancreaticobiliary maljunction 2 . More attention should be paid to pancreatic ESWL fistulas.

Endoscopy_UCTN_Code_CPL_1AK_2AF

## Notes

Endoscopy E-Videos https://eref.thieme.de/e-videos
*E-Videos* is an open access online section of the journal *Endoscopy* , reporting on interesting cases and new techniques in gastroenterological endoscopy. All papers include a high-quality video and are published with a Creative Commons CC-BY license. Endoscopy E-Videos qualify for HINARI discounts and waivers and eligibility is automatically checked during the submission process. We grant 100% waivers to articles whose corresponding authors are based in Group A countries and 50% waivers to those who are based in Group B countries as classified by Research4Life (see: https://www.research4life.org/access/eligibility/ ). This section has its own submission website at https://mc.manuscriptcentral.com/e-videos .
